# Hematologic and coagulopathy parameter as a survival predictor among moderate to severe COVID-19 patients in non- ICU ward: a single-center study at the main referral hospital in Surabaya, East Java, Indonesia

**DOI:** 10.12688/f1000research.53803.3

**Published:** 2021-11-29

**Authors:** Siprianus Ugroseno Yudho Bintoro, Ni Made Intan Dwijayanti, Dana Pramudya, Putu Niken Amrita, Pradana Zaky Romadhon, Tri Pudy Asmarawati, Arief Bachtiar, Usman Hadi

**Affiliations:** 1 Hematology – Medical Oncology Division, Department of Internal Medicine, Airlangga University, Surabaya, East Java, 60132, Indonesia; 2 Dr. Soetomo General Teaching Hospital, Surabaya, East Java, 60286, Indonesia; 3 Tropical and Infectious Diseases Division, Department of Internal Medicine, Airlangga University, Surabaya, East Java, 60132, Indonesia; 4 Department of Pulmonology and Respiratory Medicine, Airlangga University, Surabaya, East Java, 60132, Indonesia

**Keywords:** COVID-19, comorbid, hematology, coagulopathy, good health, and well-being

## Abstract

**Background :** This research aimed to examine and analyze risk factors for death, hematologic parameters and coagulation in COVID-19 patients at RSUD Dr. Soetomo Surabaya, one of the referral centers for probable COVID-19 patient cases in East Java.

**Method :** This was a retrospective analytical study by taking secondary data on patients with probable COVID-19 cases who were treated in hospital isolation rooms from May to September, 2020.

**Result : **Of 538 probable COVID-19 patients, 217 were tested positive, with an average age of 52.11±13.12 years, and there were 38 death cases. Hematologic parameters, such as white blood cell, neutrophil and lymphocyte counts, showed significantly different result in the deceased group. On the other hand, coagulation parameters, consisting of D-dimer, CRP, PT, and aPTT showed significantly similar value in the deceased group. Univariate analysis concluded that chronic kidney disease, diabetes mellitus, coronary heart disease, WBC, NLR, and PPT counts could predict the mortality, while multivariate analysis revealed that coronary heart disease was the only significant independent predictor of mortality.

**Conclusion :** This research shows that hematologic and coagulation parameters increased in the majority of COVID-19 patients and the deceased group.  While the number of neutrophils and WBC increases, the number of lymphocytes decreases significantly as the disease gets more severe.. Coronary heart disease is an independent predictor of mortality.

## Introduction

In December 2019, China reported a mysterious pneumonia case of unknown cause which had spread rapidly in Wuhan city. The World Health Organization (WHO) named this virus as the 2019 novel coronavirus (2019-nCoV), ^1^^,^^2^ and the name was changed to Severe Acute Respiratory Syndrome Coronavirus-2 (SARS-CoV-2) by the Coronaviridae Study Group (CSG) of the International Committee on Taxonomy of Viruses; the official name of the disease caused by the virus is COVID-19. ^2^^,^^3^ COVID-19 is a major health concern at this time, especially for the elderly, due to the SARS-CoV-2 virus. ^4^^,^^5^ This coronavirus has become the main pathogen, causing an outbreak of respiratory disease until it has been declared a pandemic, and spreading rapidly throughout the world, including Indonesia. ^6^ COVID-19 has become a global problem today due to the high transmission and mortality rates. ^7^

As reported by Huang *et al.*, patients with COVID-19 present primarily with fever, myalgia or fatigue, and dry cough. ^7^ Although most patients are considered to have good prognoses, elderly patients, as well as those with underlying chronic conditions, may have worse outcomes. Severe patients may experience shortness of breath and hypoxemia within one week of disease onset, which can rapidly progress to acute respiratory distress syndrome (ARDS) or end-organ damage. Chronic cardiac and metabolic disease, the presence of acute inflammation as well as decreased organ (heart, kidney, liver, and hematology) function experienced by patients at the beginning of treatment, can increase the risk of death due to COVID-19 infection. ^8^^,^^9^

The need for COVID 19 patients to be hospitalized varies widely from country to country as it depends on the prevalence of community testing and admission criteria. ^2^^,^^10^ However, it is estimated that one in 5–10 adult patients with disease severity and sufficient criteria to be hospitalized. Most of the patients with severe acute respiratory infections or severe acute respiratory syndrome were managed according to the case definition of WHO. The criteria for intensive care also vary from country to country. Old age, chronic disease, and male gender consistently associated with increased mortality. ^10^

Hematologic and coagulation parameters were important for predicting the severity of COVID-19. The occurrence of disseminated intravascular coagulation (DIC) is a common finding in COVID-19 patients. ^11^ In addition, administering anticoagulant therapy to high-risk patients is effective in reducing mortality. ^12^^,^^13^

The first COVID-19 case in Indonesia was announced on March 2, 2020, four months after the first case in China. ^14^ The first cases in Indonesia in March 2020 were two cases; and after that, two cases were noted on March 6th. COVID-19 cases continue to grow. In the beginning, there were hundreds of cases added; and until now, the number of cases has increased to thousands. On March 17, 2020, the government of East Java reported the first case of COVID-19, and as of July 31, 2020, there were 22,098 confirmed cases with a fairly high mortality rate of 7.6%. ^16^ Meanwhile, in July 2020, Surabaya reported 8,691 confirmed COVID-19 patients. The RSUD Dr. Soetomo (RSDS) Surabaya is one of the referral centres for probable COVID-19 patient cases in East Java. Based on the total cases, it is necessary to collect data, including clinical manifestations, risk factors, hematologic parameters, and coagulation which aggravate the condition of COVID-19 patients. ^17^

Several studies related to clinical manifestations and risk factors for COVID-19 patients have been reported previously; however, this research is based on relatively small sample size, and the risk factors that lead to poor clinical outcomes have not yet been well explained. In addition, probable and confirmed cases of COVID-19 at RSDS Surabaya had often presented with comorbidities, such as diabetes mellitus, high blood pressure, heart disease, hematological disorders, old age, chronic lung disease, stroke, and kidney disorders. This leads to discussion about which comorbid, hematologic parameters and coagulation factors may become predictive of COVID-19 mortality.

## Methods

This was a retrospective analytical study, performed by taking secondary data on patients with probable COVID-19 cases who were treated at the special isolation room (non-intensive care) of the Department of Internal Medicine of the teaching hospital of RSUD Dr. Soetomo between May and September, 2020. The sample consisted of deceased and survived patients at the special isolation room (non-intensive care) of Internal Medicine of RSUD Dr. Soetomo, who had been hospitalized with probable COVID-19. Incusion criteria probable COVID-19 patients were aged more than 18 years old, showing clinical signs of pnemoniae. They are categorized into moderate pneumoniae with signs and symptoms as follows: fever, cough, dyspnea, tachypnea, and later grouped into the severe category with additional criteria, such as: RR > 30 times per minute, O _2_ sat <93% room air, also no mechanical support and ICU care needed. Patients with incomplete data were excluded.

**Figure 1.**
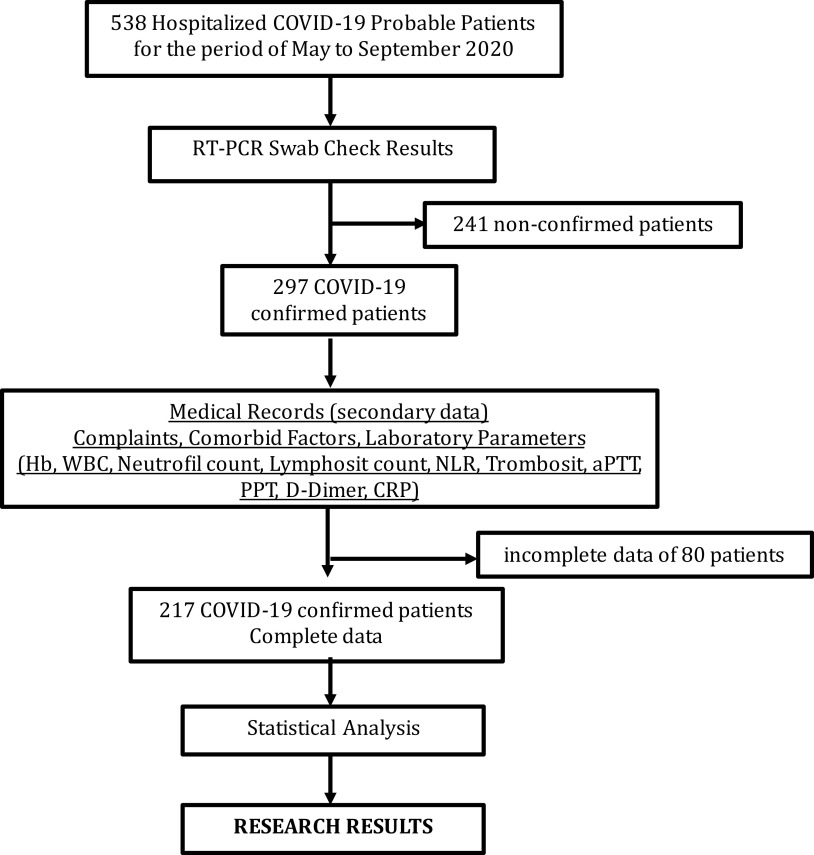
Patient COVID-19 data selection process.

According to hospitalized probable COVID-19 criteria, there were 538 probable cases. COVID-19 was diagnosed through sample taken from nasal swab and further RT PCR examination. 297 patients showed positive result, whereas the rest are negative. Of 297 patients, 80 had incomplete medical record, hence, 217 samples left eventually were included (see Figure 1).

Approval from the local ethics committee was obtained for this research; written informed consent was obtained from patients during hospitalization. From the hospital infromation database system, we retrieved patient characteristics such as age, gender, comorbidities, signs and symptoms, and laboratory results, including hemoglobin, white blood cell (WBC), neutrophil, lymphocyte, and platelet counts, D-dimer level, C-reactive protein (CRP), prothrombin time (PT), and activated partial thromboplastin time (aPTT).

### Statistical analysis

Data analysis was performed using SPSS version 25 (Chicago, IL, USA; RRID:SCR_002865); JASP ( RRID:SCR_015823) is an open access alternative. Patient characteristics (see Table 1) are presented as mean ± standard deviation or median, and interquartile ranges of 25 ^th^ and 75 ^th^ percentiles (IQR 1–3) or minimum and maximum, depending on the continuous distribution variable. Normality tests were performed and a comparison test for normal distribution data by an independent sample t-test and a Mann–Whitney test, otherwise. We used ANOVA to determine statistical differences among some groups with normally-distributed data. contrarily, we carried out Kruskal Wallis analysis in groups with abnormally distributed ones. Comparison for the caterogical variables were performed using Pearson's and Fisher's Exact Chi-squared tests. Survival analyses and Kaplan–Meier survival curves were performed for hemoglobin, white blood cell, neutrophil, lymphocyte, neutrophil–lymphocyte ratio, D-dimer, PT, aPTT, and CRP.

**Table 1.** Laboratory and clinical overview of the deceased and survived patients.

	Total (n = 217)	Survival status		p-value
		Not survive (n = 38)	Survive (n = 179)	
Age	52.11 ± 13.12	58.42 ± 12.78	50.77 ± 12.83	**<0.001**
Gender				
Male	116 (53.5)	23 (60.5)	93 (52.0)	0.336
Female	101 (46.5)	15 (39.5)	86 (48.0)	
Comorbid factors	129 (59.4)	28 (73.7)	101 (56.4)	**0.049**
Diabetes (DM)	72 (33.2)	18 (47.4)	54 (30.2)	**0.041**
Hypertension (HT)	66 (30.4)	13 (34.2)	53 (29.6)	0.575
Coronary heart disease	4 (1.8)	4 (10.5)	0 (0)	**<0.001** *
Thyroid	2 (0.9)	0 (0)	2 (1.1)	0.680 *
Obesity	2 (0.9)	0 (0)	2 (1.1)	0.680 *
Malignancy	10 (4.6)	4 (10.5)	6 (3.4)	0.076 *
CKD	42 (19.4)	27 (15.1)	15 (39.5)	**<0.001**
Regular HD	11 (5.1)	9 (5.0)	2 (5.3)	0.605 *
Hepatitis B	7 (3.2)	4 (2.2)	3 (7.9)	0.105 *
Chronic liver disease	5 (2.3)	3 (1.7)	2 (5.3)	0.211 *
Chronic lung disease	5 (2.3)	0 (0)	5 (2.8)	0.378 *
Symptoms				
Short of breath	118 (54.4)	25 (65.8)	93 (52.0)	0.120
Cough	111 (51.2)	19 (50.0)	92 (52.4)	0.876
Fever	87 (40.1)	19 (50.0)	68 (38.0)	0.170
Limp	51 (23.5)	11 (28.9)	40 (22.3)	0.383
Hoarseness	0 (0)	0 (0)	0 (0)	-
Anosmia	2 (0.9)	0 (0)	2 (1.1)	0.680 *
Nasal congestion	1 (0.5)	0 (0)	1 (0.6)	0.825 *
Watery eyes	0 (0)	0 (0)	0 (0)	-
Muscle pain	0 (0)	0 (0)	0 (0)	-
Diarrhea	25 (11.5)	4 (10.5)	21 (11.7)	0.833
Swallowing pain	9 (4.1)	2 (5.3)	7 (3.9)	0.705
Headache	5 (2.3)	1 (2.6)	4 (2.2)	0.883
COVID-19 therapies				
Hydroxychloroquine	35 (16.1)	2 (5.3)	33 (18.4)	-
Isoprinosine	25 (11.5)	6 (15.8)	19 (10.6)	-
Oseltamivir	14 (6.5)	13 (7.3)	1 (2.6)	-
Lopinavir	38 (17.5)	11 (28.9)	27 (15.1)	-
Favipiravir (Avigan)	2 (0.9)	0 (0)	2 (1.1)	-
Laboratory parameters				
Hb (g/dL)	12.27 ± 2.54	11.75 ± 2.30	12.38 ± 2.58	0.075 **
WBC (×10 ^9^/L)	10.83 ± 9.64	13.67 ± 11.83	10.23 ± 9.03	**0.011** **
Neutrophil abs (×10 ^9^/L)	8.19 ± 7.39	13.67 ± 11.13	10.23 ± 6.16	**0.002** **
Lymphocyte abs (×10 ^9^/L)	1.46 ± 1.34	1.39 ± 1.54	1.48 ± 0.11	0.074 **
NLR	8.53 ± 17.87	15.57 ± 39.14	7.04 ± 7.51	**0.003** **
PLT count (×10 ^9^/L)	298.87 ± 174.02	315.28 ± 253.73	295.39 ± 152.63	0.953 **
aPTT (second)	28.82 ± 9.14	31.37 ± 17.40	28.28 ± 6.07	0.635 **
PPT (second)	12.58 ± 6.81	15.06 ± 14.27	12.06 ± 3.52	0.103 **
D-Dimer (ng/dL)	3593.15 ± 5380.45	6767.55 ± 8655.18	2919.26 ± 4117.03	**<0.001** **
CRP (mg/dL)	12.95 ± 45.54	17.80 ± 11.55	11.92 ± 49.83	**<0.001** **

* Fischer Exact test.

** Mann-Whitney test.

The evaluation of independent predictors of mortality was perfomed by univariate and multivariate Cox regression analysis. Threshold mortality predictor from the laboratory parameter was also performed; the receiver–operating curve (ROC) analysis associated with the area under the curve (AUC) was used to find the optimal threshold value of the laboratory rate parameter to predict the progression of mortality in the study group. The AUC was interpreted as excellent if 0.9 < AUC < 1; good if 0.8 < AUC < 0.9; moderate if 0.7 < AUC < 0.8; poor if 0.6 < AUC < 0.7; and fail if 0.5 < AUC < 0.6.

## Results

538 patients with probable COVID-19 were included during the study period in which. 288 (53.5%) were males, with the mean ± SD age of 51.69 ± 13.67 years. More than half of the patients (63.7%) had elemental diseases, including diabetes mellitus (34%), hypertension (32.5%), and CKD (22.7%). All the patients with probable COVID-19 experienced symptoms on arrival, including complaints of shortness of breath (48.7%), cough (40%), fever (32%), and limp (24%). Some patients also complained of diarrhea and headache but it was only at 10.2% and 3.2%, respectively.

### Demographic and clinical overview and laboratory results of the COVID-19 patient study group

In total, we included 217 hospitalized patients with a diagnosis of COVID-19; they were 116 male patients and the male-to-female ratio was 1.14. The average age of this retrospective research was 52.11 ± 13.12 years. The most common chronic disease (comorbid) among patients was diabetes (33.2%). The clinical and demographic characteristics and laboratory results are shown in Table 1.

### Comparison of laboratory parameters in deceased and survived patients

From 217 patients, 38 patients (17.5%) died. They were significantly older than those who survived (mean 58.42 ± 12.78 *versus* 50.77 ± 12.83 years, respectively; p < 0.001). The majority of the deceased patients had at least one comorbidity (73%), while 56.4% of the patients who survived had comorbid factors (p = 0.049). The common comorbidities that were significantly different were diabetes mellitus, hypertension, and coronary heart disease in the group of deceased patients than in those who survived. We stratified laboratory markers that we divided based on normal lab values. In deceased patients, the white blood cell, neutrophil, and lymphocyte counts showed significant difference between groups. Otherwise, D-dimer, CRP, PPT and aPTT did not show any differences (see Table 2).

**Table 2.** Comparison of laboratory results in deceased and survived patients.

	Total (n = 217)	Survival status		p-value
		Not survive (n = 38)	Survive (n = 179)	
Hemoglobin (g/dL) (%)				
<12	78 (35.9)	20 (52.6)	58 (52.4)	0.052 *
12-16	136 (62.7)	18 (47.4)	118 (65.9)	
>16	3 (1.4)	0 (0)	3 (1.7)	
WBC (leucocyte) (×10 ^9^) (%)				
<4.0	13 (6.0)	2 (5.3)	11 (6.1)	**0.028**
4.0-10.0	125 (57.6)	15 (39.5)	110 (61.5)	
>10.0	79 (36.4)	21 (55.3)	28 (32.4)	
Neutrophil count (×10 ^9^) (%)				
<2.0	9 (4.1)	2 (5.3)	7 (3.9)	**0.006**
2.0-7.0	119 (54.8)	12 (31.6)	107 (59.8)	
>7.0	89 (41.0)	24 (63.2)	65 (36.3)	
Lymphocyte count (×10 ^9^) (%)				
<0.8	47 (21.7)	12 (31.6)	35 (19.6)	**0.048** *
0.8-4.0	166 (76.5)	24 (63.2)	142 (79.3)	
>4.0	4 (1.8)	2 (5.3)	2 (1.1)	
Platelet count (×10 ^9^) (%)				
<150	27 (12.4)	3 (7.9)	24 (13.4)	0.588 *
150-450	164 (75.6)	31 (81.6)	133 (74.3)	
>450	26 (12.0)	4 (10.5)	22 (12.3)	
Above-normal result percentages				
C-reactive protein (%)	192 (88.5)	37 (97.4)	155 (86.6)	**0.042**
D-dimer (%)	203 (93.5)	36 (94.7)	167 (93.3)	0.543
Prothrombin time (%)	12 (5.5)	5 (13.2)	7 (3.9)	**0.039**
aPTT (%)	7 (3.2)	2 (5.3)	5 (2.8)	0.354
NLR	154 (71)	33 (86.8)	121 (67.6)	**0.018**

* Kruskal-Wallis test.

Neutrophil-lymphocyte ratio (NLR), Lactate dehydrogenase (LDH), International normalized ratio (INR), Activated partial thromboplastin time (aPTT). Normal laboratory reference values: CRP: 0–5mg /L, D-dimer: 0–500 ng/mL, PT: 11–16 seconds, aPTT: 25–40 seconds.

Inflammatory markers (leukocyte, C-reactive protein, neutrophill count and NLR) were significantly higher in the deceased group than in the group who survived. While the normal leukocyte and neutrophil counts were significantly more common in the patients who survived, and lymphopenia was significantly more common in deceased patients (p = 0.048). Meanwhile, the frequency of thrombocytopenia and the increase in D-dimer were comparable between two groups. Neutropenia, leukopenia, lymphopenia, higher CRP, NLR, and PT correlated with COVID-19 patients’ mortality.

The ROC analysis using sensitivity and specificity based on mortality predictor revealed that there was an optimal cut-off value for several laboratory parameters including WBC count, neutrophil count, CRP level, D-dimer, and NLR count. The largest AUC value was CRP with a cut-off value of ≥1.85 (sensitivity = 94.7% and specificity = 72.1%). NLR, WBC count, neutrophil count, D-dimer, and CRP as mortality predictors showed good results (see Table 3).

**Table 3.** Sensitivity and specificity of laboratory parameters.

	AUC	Sensitivity	Specificity	Cut-off	95% CI	p-value
Hemoglobin (g/dL)	0.408	0.782	0.658	>10.85	0.311-0.505	0.075
WBC (×10 ^9^)	0.631	0.620	0.737	≥7090	0.534-0.728	**0.011**
Neutrophil count (×10 ^9^)	0.663	0.961	0.947	≥2080	0.570-0.756	**0.002**
Lymphocyte count (×10 ^9^)	0.408	0.765	0.632	≥859	0.306-0.509	0.074
Platelet count (×10 ^9^)	0.497	0.687	0.684	≥208000	0.401-0.593	0.953
C-reactive protein	0.773	0.947	0.721	≥1.85	0.693-0.852	**<0.001**
D-dimer	0.679	0.939	0.974	≥410	0.586-0.772	**<0.001**
Prothrombin time	0.584	0.877	0.895	≥9.95	0.476-0.692	0.103
aPTT	0.525	0.385	0.421	≥29	0.428-0.621	0.635
NLR	0.652	0.592	0.789	≥3.88	0.563-0.743	**0.003**

### Survival analysis

**Figure 2.**
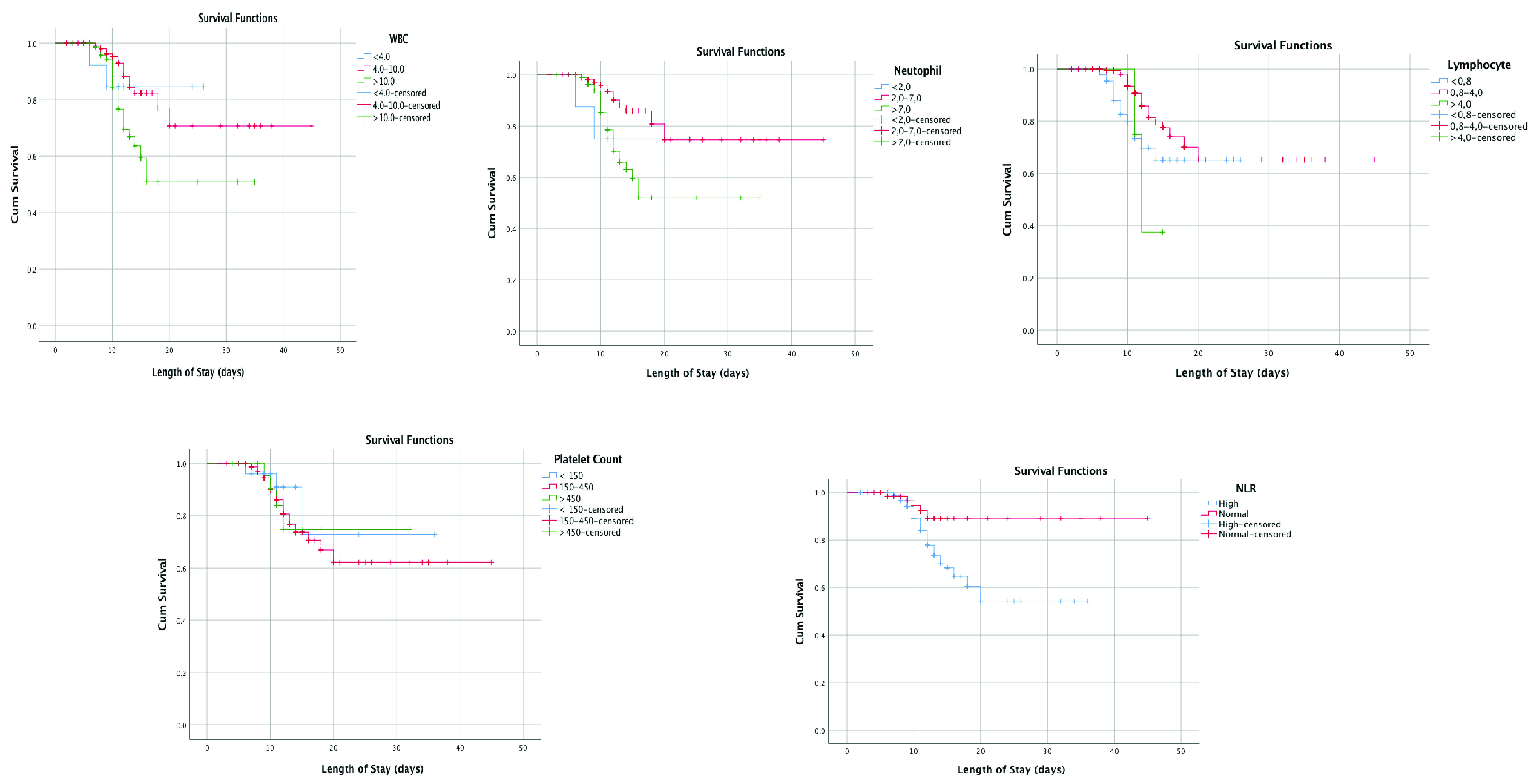

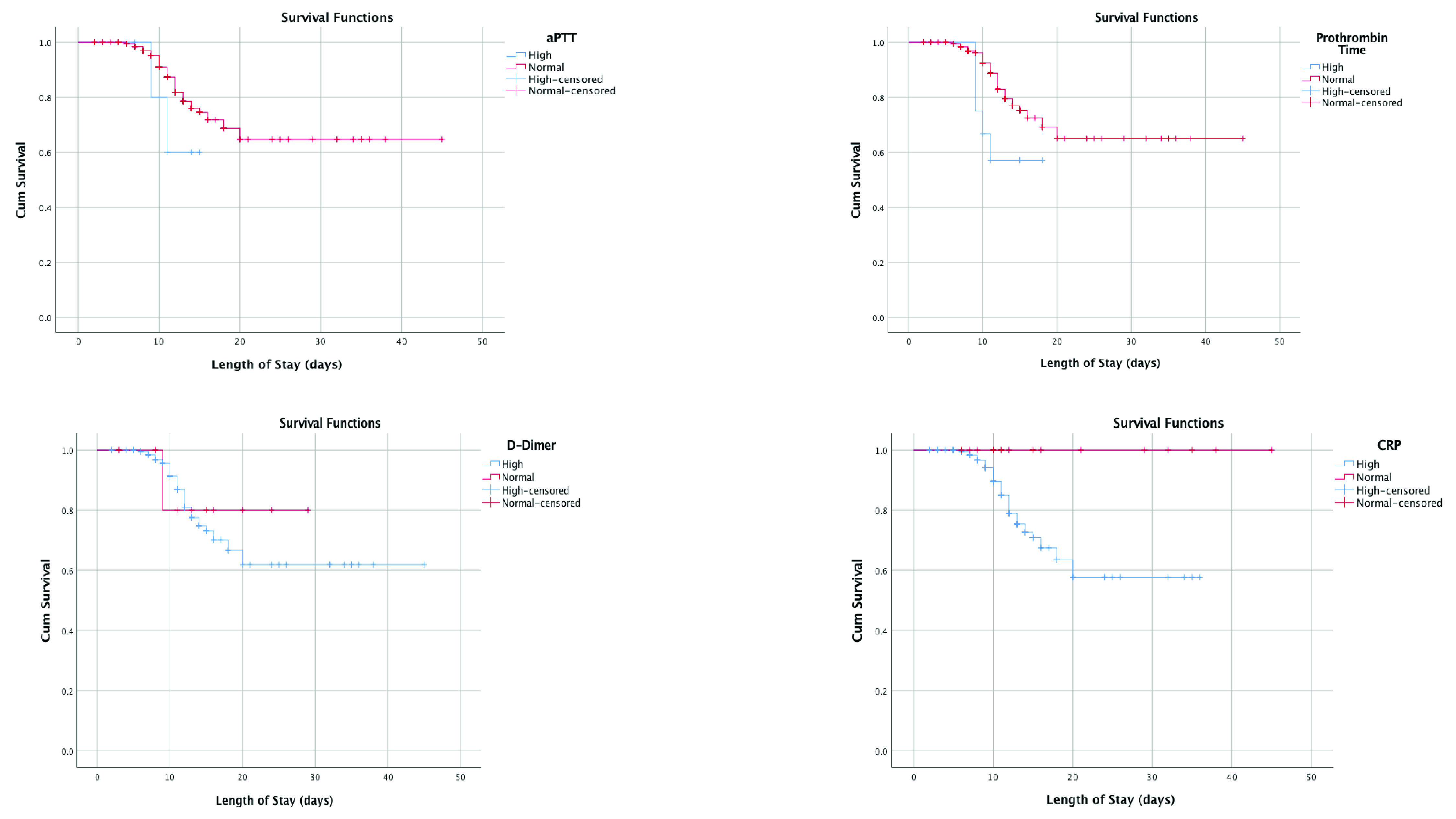
Kaplan-Meier survival curves white blood cell count (WBC), neutrophil count, lymphocyte count, neutrophil-lymphocyte ratio (NLR), platelet count, aPTT, prothrombin time, D-Dimer, and CRP.

The Kaplan–Meier graph showed that leukocyte count, neutrophil count, NLR, PT, and CRP associated with patients mortality, that they can be used to predict it (p < 0.05, respectively) (see Figure 2). Moreover, lower survival rate was shown from leukopenia, leukocytosis, neutrophillia, high NLR, high CRP, and prolonged PT (p = 0.015; p = 0.018, p = 0.003, p = 0.035, and p = 0.03, respectively).

### Determining independent predictors of mortality

We included age (as a categorical variable of under and over 65 years old), presence of chronic kidney disease, diabetes mellitus, coronary heart disease, and indicators, such as WBC count, NLR, CRP, PT, aPTT, and D-dimer for determining the predictors of mortality. In the univariate analysis, the independent predictors of mortality were shown from presence of chronic kidney disease, diabetes mellitus, coronary heart disease, WBC count, NLR, and PPT. However, based on the Cox multivariate regression analysis, only coronary heart disease significantly became the independent predictor of mortality (see Table 4).

**Table 4.** Multivariate with Cox regression analysis.

	Crude HR (95% CI)	Crude p-value	Adjusted HR (95% CI)	Adjusted p-value
Age >65 years old: exists or not	2.05 (0.99-4.23)	0.051		
Hypertension: exists or not	1.16 (0.59-2.28)	0.647		
CKD: exists or not	2.76 (1.44-5.29)	**0.002**	1.64 (0.78-3.41)	0.185
Diabetes mellitus: exists or not	1.95 (1.03-3.71)	**0.039**	1.69 (0.84-3.39)	0.136
Coronary heart disease: exists or not	9.24 (3.20-26.67)	**<0.001**	11.56 (3.24-41.26)	**<0.001**
Platelet count: high or normal	0.971 (0.34-2.74)	0.956		
WBC count: high or normal	2.47 (1.29-4.69)	**0.006**	1.58 (0.76-3.29)	0.216
NLR: high or normal	2.91 (1.13-7.47)	**0.026**	1.81 (0.66-4.98)	0.247
C-reactive protein: high or normal	6.31 (0.86-46.30)	0.070		
Prothrombin time: high or normal	2.68 (1.04-6.91)	**0.040**	1.91 (0.67-5.39)	0.222
aPTT: high or normal	2.24 (0.53-9.34)	0.268		
D-Dimer	1.42 (0.34-5.93	0.630		

## Discussion

Significant differences were found in the demographic and clinical variables, and hematologic and coagulation parameters between the deceased and surviving COVID-19 patients. We included age in the risk factor for COVID-19 mortality, whereas the age had a p-value <0.05. The age factor appeared to be crucial for the outcome of COVID-19. The average age of the deceased patients was 58 years old and was significantly older than the surviving patients. This was in accordance with previous studies that older age has been reported as an important independent predictor of mortality in SARS-Cov2. ^15^^,^^16^ Increasing age also increased the percentage of COVID-19 mortality, the age-dependent defects in T-cell and B-cell function could lead to a deficiency in control of viral replication and more prolonged pro inflammatory responses, potentially leading to poor outcome. ^17^

Gender was proven to be a risk factor for mortality in COVID-19 patients, which was higher for men than for women. This is thought to be due to differences in the immunological systems of men and women, differences in lifestyle, and the prevalence of smoking. ^18^ In this research, although statistically insignificant, the percentage of the number of male COVID-19 patients was higher, both overall and in the group of deceased patients. The higher mortality rates were associated with the higher chronic comorbidities in men, *e.g.*, diabetes mellitus, kidney disease, hypertension, heart disease, lung disease, and smoking. ^19^

The comorbid factors of diabetes mellitus, heart disease, and chronic kidney disease in COVID-19 patients could be the risk factors of death in this research, with a p-value of <0.05. Previous studies have described that the presence of common comorbidities increase COVID-19 patients risk. This result was similar to the meta-analysis study conducted by Mantovani *et al.*, who stated that the prevalence of diabetic patients hospitalized due to COVID-19 was 14.34%, and 11.06% in patients in Asian countries. Meanwhile, the prevalence in non-Asian countries was higher, which was 23.34%. The risk of worsening the condition to require treatment in hospital was greater in COVID-19 patients with pre-existing diabetes. ^20^ However, the specific comorbidity by which can lead to disease progression remain unknown in COVID-19 patients. ^21^

For the hematologic parameters in this research, the leukocyte, neutrophil counts, lymphocyte count, and NLR demonstrated significantly different result between two groups. This research results were consistent with several previously published studies. ^22^^,^^23^ On the other hand, the platelet count in this research was comparable between the groups of deceased and surviving patients. This was in contrast with the results of a meta-analysis that concluded by Lippi *et al.*, who showed that thrombocytopenia was associated with increasing severity risk and mortality of COVID-19. ^24^ Differences in pathophysiological mechanisms in each patient may lead to insignificant findings in this research. Many researchers have studied the changes in peripheral blood cell counts in COVID-19, and the results were that in infected patients, the white blood cell and neutrophil count increased, otherwise, the lymphocyte and platelet counts decreased. ^25^ In the other cases, coagulation abnormalities (prolonged PT and aPTT) and intravascular coagulopathy (DIC) were so correlated with low platelet count. ^26^

The extreme inflammation is usually evidenced by elevated serum of CRP, IL-6, and PCT which indicate the increasing of COVID-19 severity. ^27^ High levels of CRP and procalcitonin in COVID-19 patients also associated with the progression of ARDS, myocardial injury, and death. ^27^^,^^28^ The presence of secondary bacterial infection would be an additional explanation of this increase in inflammatory biomarkers. This is consistent with this research which showed that serum CRP levels were significantly higher in the cohort of deceased COVID-19 patients. Therefore, we believe that the use of CRP as a biomarker in monitoring the progress and severity of COVID-19 patients will be considerably beneficial.

D Dimer in our study were measured using ELFA (enzyme linked fluorescent assay) method and cut of point 0.410 µg/ml. Several literatures showed different cut off point for D Dimer. Poudel, 2021 with 182 subject research showed D Dimer level 1.5 µg/ml were an optimal cut off point to predict mortality. ^29^ While Zhang, 2020 with 343 subjects shows D Dimer level 2 µg/ml were an optimal cut off point. ^30^ Guan, 2020 in China with 1099 subjects showed that non-survivors had a significantly higher D-dimer (median: 2.12 μg/ml) than that of survivors (median: 0.61 μg/ml). ^31^ This result could be due to differences in measurement methods as disclosed by Favaloro et al. who stated several things regarding the measurement and reporting quality of D-dimers such as the measurement method, cut-off value, or D-dimer unit [D-dimer unit (DDU)] can lead to different research results. ^32^ Other factors in our study that can affect D dimer level were our patients were older with median age 52, comorbidities such as cardiovascular disease and liver disease. Liver disease can affects D Dimer elimination from blood. In this study D dimer level was also measured on admission, very early in disease course. The half-life of D-dimer was approximately 8 hours therefore, serial measurement of D-dimer will shows more information.

PT and aPTT prolongation may occur during severe COVID-19, yet the increase is not as high as what has been observed in bacterial sepsis and DIC. A meta-analysis conducted by Henry *et al.* ^25^ found that patients with severe and fatal COVID-19 had significantly higher coagulation parameters (especially PT) than patients with the non-severe ones. This is consistent with this research where there was an increase in aPTT, albeit insignificant, and a significant increase in PT with p < 0.05 in the group of deceased patients. Although it is not completely clear how SARS-CoV-2 activates the coagulation cascade, it may be associated as a by-product of cytokine storms. ^33^ Researchers detected a significant extension in coagulation tests in this research, consistent with previously published studies. ^25^^,^^34^ The mechanism of these changes is still not fully explained, however, the extension of the coagulation test, *i.e.*, increased PT and aPTT, can be considered as a marker of disease severity and activation of the coagulation cascade and virus-induced cytokine storm. ^33^^,^^35^

For as much as this research was a retrospective study, several parametes were not completely listed such as level of ferritin, fibrinogen, procalcitonin, and IL-6, that we considered this as one of our research limitations. Since the development of complications that have occured in patients are not thoroughly documented, we could not confidently say that inflammatory and coagulation factor disorders are more frequent in severe COVID-19. The only outcome measure of this research was patient mortality in hospital. Although researchers evaluated platelet count and D-dimers, the International Society on Thrombosis and Hemostasis (ISTH) scores of the patients were not calculated, thus patients who had mild-to-moderate coagulation disorders could not be distinguished from who had a DIC.

## Conclusion

This research indicates that hematologic and coagulation parameters are increased in the majority of COVID-19 patients and the group of deceased patients. As the neutrophil count and WBC increase, the lymphocyte count declines significantly along with the increase in disease severity. Coronary heart disease is an independent predictor of mortality.

## Data availability

### Underlying data

Figshare: Underlying data for ‘Hematologic and coagulopathy parameter as a survival predictor among moderate to severe COVID-19 patients in non- ICU ward: a single-center study at the main referral hospital in Surabaya, East Java, Indonesia’. https://doi.org/10.6084/m9.figshare.14673060.

The project contains the following underlying data:

• Hema_Coagul_parameter_COVID.xlsx (main data).
• readme.docx (index).

Data are available under the terms of the Creative Commons Attribution 4.0 International license (CC-BY 4.0).

## Consent

Written informed consent was received from the patients during hospitalization.
