# Supplementary material for: Hematologic and coagulopathy parameter as a survival predictor among moderate to severe COVID-19 patients in non- ICU ward: a single-center study at the main referral hospital in Surabaya, East Java, Indonesia
Source: F1000Res. 2021 Nov 29;10:791. Originally published 2021 Aug 11. [Version 3] doi: 10.12688/f1000research.53803.3 (PMC8596187; doi:10.12688/f1000research.53803.3)
Supplement: Peer review history

# Peer review: Reviewer response for version 3

**DOI:** 10.5256/f1000research.79610.r101465

**Published:** 2021-12-01

**Authors:** Simona Lattanzi

**Affiliations:** 1 Neurological Clinic, Department of Experimental and Clinical Medicine, Marche Polytechnic University, Ancona, Italy

The Authors did not address any of the issues raised in my previous report. The issues should be considered in revising the manuscript.

Is the work clearly and accurately presented and does it cite the current literature?

Yes

If applicable, is the statistical analysis and its interpretation appropriate?

Yes

Are all the source data underlying the results available to ensure full reproducibility?

Yes

Is the study design appropriate and is the work technically sound?

Yes

Are the conclusions drawn adequately supported by the results?

Yes

Are sufficient details of methods and analysis provided to allow replication by others?

Yes

Reviewer Expertise:

Neurology

I confirm that I have read this submission and believe that I have an appropriate level of expertise to confirm that it is of an acceptable scientific standard, however I have significant reservations, as outlined above.

# Peer review: Reviewer response for version 3

**DOI:** 10.5256/f1000research.79610.r101467

**Published:** 2021-11-30

**Authors:** Azlan Bin Husin

**Affiliations:** 1 Hospital Universiti Sains Malaysia, Kubang Kerian, Malaysia; 2 School of Medical Sciences, Universiti Sains Malaysia, Kubang Kerian, Malaysia

Congratulations, all comments has been addressed well.

Is the work clearly and accurately presented and does it cite the current literature?

Partly

If applicable, is the statistical analysis and its interpretation appropriate?

Yes

Are all the source data underlying the results available to ensure full reproducibility?

No

Is the study design appropriate and is the work technically sound?

Yes

Are the conclusions drawn adequately supported by the results?

No

Are sufficient details of methods and analysis provided to allow replication by others?

No

Reviewer Expertise:

Clinical hematology, benign hematology, malignant hematology, autologous hematopoietic stem cell transplant

I confirm that I have read this submission and believe that I have an appropriate level of expertise to confirm that it is of an acceptable scientific standard.

# Peer review: Reviewer response for version 2

**DOI:** 10.5256/f1000research.77444.r100136

**Published:** 2021-11-16

**Authors:** Simona Lattanzi

**Affiliations:** 1 Neurological Clinic, Department of Experimental and Clinical Medicine, Marche Polytechnic University, Ancona, Italy

This was a retrospective study aiming to evaluate the clinical and laboratory predictors of mortality in COVID-19 patients.

 The study is interesting and provides useful information in clinical practice. There are, however, some issues that need to be further addressed.

 Were levels of interleukins available? Could you explore any correlations with the other identified predictors?

 There is increasing evidence that easily obtainable laboratory biomarkers can have a predictive role and be used for diagnosis and outcome prediction in a variety of medical conditions not limited to infectious pathologies, but also including major cardiac events (ref 1), cerebral hemorrhage (ref 2), ischemic stroke (ref 3; ref 4), and cancers (ref 5; ref 6). In light of the suggested evidence, please put the study findings into the current research context by adding a brief synthesis about the meaning and wide potentialities of easy to obtain and inexpensive serum biomarkers in everyday clinical practice.

Is the work clearly and accurately presented and does it cite the current literature?

Yes

If applicable, is the statistical analysis and its interpretation appropriate?

Yes

Are all the source data underlying the results available to ensure full reproducibility?

Yes

Is the study design appropriate and is the work technically sound?

Yes

Are the conclusions drawn adequately supported by the results?

Yes

Are sufficient details of methods and analysis provided to allow replication by others?

Yes

Reviewer Expertise:

Neurology

I confirm that I have read this submission and believe that I have an appropriate level of expertise to confirm that it is of an acceptable scientific standard, however I have significant reservations, as outlined above.

## References

[1] : Association between admission neutrophil to lymphocyte ratio and outcomes in patients with acute coronary syndrome. *Am J Cardiol* .2008;102(6) : 10.1016/j.amjcard.2008.05.006 653-7 10.1016/j.amjcard.2008.05.006 18773982

[2] : Neutrophil-to-lymphocyte ratio improves outcome prediction of acute intracerebral hemorrhage. *Journal of the Neurological Sciences* .2018;387: 10.1016/j.jns.2018.01.038 98-102 10.1016/j.jns.2018.01.038 29571881

[3] : Systemic Inflammatory Response Index and Futile Recanalization in Patients with Ischemic Stroke Undergoing Endovascular Treatment. *Brain Sci* .2021;11(9) : 10.3390/brainsci11091164 10.3390/brainsci11091164 34573185PMC8468021

[4] : Neutrophil-to-Lymphocyte Ratio and Symptomatic Hemorrhagic Transformation in Ischemic Stroke Patients Undergoing Revascularization. *Brain Sci* .2020;10(11) : 10.3390/brainsci10110771 10.3390/brainsci10110771 33114150PMC7690817

[5] : Exploring the prognostic value of the neutrophil-to-lymphocyte ratio in cancer. *Scientific Reports* .2019;9(1) : 10.1038/s41598-019-56218-z 10.1038/s41598-019-56218-z 31873162PMC6928022

[6] : The systemic immune-inflammation index is an independent predictor of survival for metastatic colorectal cancer and its association with the lymphocytic response to the tumor. *Journal of Translational Medicine* .2018;16(1) : 10.1186/s12967-018-1638-9 10.1186/s12967-018-1638-9 30286769PMC6172841

# Peer review: Reviewer response for version 2

**DOI:** 10.5256/f1000research.77444.r94904

**Published:** 2021-09-28

**Authors:** Andhika Rachman, Bayu Rumondor

**Affiliations:** 1 Department of Hematology and Medical Oncology, University of Indonesia, Jakarta, Indonesia; 2 Kebayoran Baru General Hospital Jakarta, South Jakarta, Indonesia

There are no further comments. Thank you for the revision

Is the work clearly and accurately presented and does it cite the current literature?

Yes

If applicable, is the statistical analysis and its interpretation appropriate?

Yes

Are all the source data underlying the results available to ensure full reproducibility?

Partly

Is the study design appropriate and is the work technically sound?

Partly

Are the conclusions drawn adequately supported by the results?

Yes

Are sufficient details of methods and analysis provided to allow replication by others?

Partly

Reviewer Expertise:

Immunology, Cancer thrombosis, and Platelets

We confirm that we have read this submission and believe that we have an appropriate level of expertise to confirm that it is of an acceptable scientific standard.

# Peer review: Reviewer response for version 1

**DOI:** 10.5256/f1000research.57221.r91683

**Published:** 2021-08-31

**Authors:** Azlan Bin Husin

**Affiliations:** 1 Hospital Universiti Sains Malaysia, Kubang Kerian, Malaysia; 2 School of Medical Sciences, Universiti Sains Malaysia, Kubang Kerian, Malaysia

1. Policy or criteria for hospital admission is important information to be included or at least discussed
2. Inclusion of patients who were not having COVID19 is not relevant in this research, unless author want to compare between positive vs negative group
3. This study used secondary data retrieved in respective manner; hence the documentation for informed consent from each patients is irrelevant
4. Operational definition for relevant co-morbidities were not available eg: thyroid disease (hypothyroid or hyperthyroid) or coronary artery disease (was it based on coronary angiogram or ECG or echocardiogram only or self declaration), etc.
5. Mortality should be defined - whether it is overall or specific of any term
6. Should look into more meaningful key points to compare between this study results with other published reports. 
  Eg: a) Regarding age: comparing mean age is better. While discussing on this point it is important to consider relevant differences in study population (eg: hospital admission criteria that may include age, presence of co-morbidity etc)
7. Some statements were too strong and not supported by cited reference e.g. paragraph 2 (regarding gender issues), paragraph 3 (on elderly and diabetes), and paragraph 4 (about pathophysiologic mechanism) of the discussion.
8. Discussion on D-dimer should be supported by comparing the method and cut off point used in this study versus with other published data. Should also look into possible confounding factors like superimposed infection or may be smaller number of patients

Is the work clearly and accurately presented and does it cite the current literature?

Partly

If applicable, is the statistical analysis and its interpretation appropriate?

Yes

Are all the source data underlying the results available to ensure full reproducibility?

No

Is the study design appropriate and is the work technically sound?

Yes

Are the conclusions drawn adequately supported by the results?

No

Are sufficient details of methods and analysis provided to allow replication by others?

No

Reviewer Expertise:

clinical hematology, benign hematology, malignant hematology, autologous hematopoietic stem cell transplant

I confirm that I have read this submission and believe that I have an appropriate level of expertise to confirm that it is of an acceptable scientific standard, however I have significant reservations, as outlined above.

# Response

**Published:** 2021-08-31

**Authors:** Siprianus Ugroseno Yudho Bintoro

**Affiliations:** Airlangga University, Indonesia

Dear reviewer,

 Thank you for reviewing and sending the feedback. We are going to revise it real soon.

 warm regards,

 Research Team

# Response

**Published:** 2021-11-18

**Authors:** Siprianus Ugroseno Yudho Bintoro

**Affiliations:** Airlangga University, Indonesia

**Comment 1:**

 Policy or criteria for hospital admission were compliant with hospital policy which are: confirmed or suspected Covid-19, who are symptomatic plus any of following criteria.

1. Age > 65 years.
2. Clinical or radiologic evidence of pneumonia.
3. O _2 _ saturation Spo _2   _< 99 %.
4. Acute respiratory distress syndrome.
5. COPD
6. CKD
7. Acute compilation of diabetes mellitus.
8. Obesity (BMI ≥ 40).
9. Acute malignancy.
10. Other to illness by physician description.

We included inclusion and exclusion  criteria in Methods paragraph 2 in the revised version.

**Comment 2:**

 We appreciate your very helpful comments. We included the probable patients to show the magnitude of Covid-19 probable cases in our referral hospital.  

**Comment 3:**

 We appreciate your very helpful comment. However we already have the informed consent to use patient’s secondary administrative during their time of admission.  

**Comment 4:**

 Thank you for your very helpful comment. Comorbidities were recorded at admission by attending clinical staff (board certified interns), either by history taking, records from previous visits in our hospital, or previous laboratory result assessment, or patient reporting. We added it in the manuscript: Methods paragraph 2

**Comment 5:**

 Thank you for your suggestion. In patients with confirmed Covid-19 we use the definition of mortality based on the WHO definition: death resulting from a clinically compatible illness in a probable or confirmed Covid-19 case, unless there is a clear alternative cause of death that cannot be related to Covid-19 (e.g trauma). There should be no periode of complete recovery between the illness and death.

**Comment 6:**

 We appreciate your comment, we revised our manuscript in Discussion, paragraphs 1 to 4.

 Significant differences were found in the demographic and clinical variables, and hematologic and coagulation parameters between the deceased and surviving COVID-19 patients. We included age in the risk factor for COVID- 19 mortality, whereas the age had a p-value <0.05. The age factor appeared to be crucial for the outcome of COVID-19. The average age of the deceased patients was 58 years old and was significantly older than the surviving patients. This was in accordance with previous studies that older age has been reported as an important independent predictor of mortality in SARS-Cov2. ^15,16^ Increasing age also increased the percentage of COVID-19 mortality, the age-dependent defects in T-cell and B-cell function could lead to a deficiency in control of viral replication and more prolonged proinflammatory responses, potentially leading to poor outcome. ^17^

**Comment 7:**

 Gender was suspected to be a risk factor for mortality in COVID-19 patients, which was higher for men than for women. This is thought to be due to differences in the immunological systems of men and women, differences in lifestyle, and the prevalence of smoking. ^18^ In this research, although statistically insignificant, the percentage of the number of male COVID-19 patients was higher, both overall and in the group of deceased patients. The higher mortality rates were associated with the higher chronic comorbidities in men, e.g., diabetes mellitus, kidney disease, hypertension, heart disease, lung disease, and smoking. ^19^

 The comorbidity factors of diabetes mellitus, heart disease, and chronic kidney disease in COVID-19 patients could be the risk factors of death in this research, with a p-value of <0.05. Previous studies have described that the presence of common comorbidities increase COVID-19 patients risk. This result was similar to the meta-analysis study conducted by Mantovani *et al.*, who stated that the prevalence of diabetic patients hospitalized due to COVID-19 was 14.34%, and 11.06% in patients in Asian countries. Meanwhile, the prevalence in non-Asian countries was higher, which was 23.34%. The risk of worsening the condition to require treatment in hospital was greater in COVID-19 patients with pre-existing diabetes. ^20^ However, the specific comorbidity by which can lead to disease progression remains unknown in COVID-19 patients. ^21^

 For the hematologic parameters in this research, the leukocyte, neutrophil counts, lymphocyte count, and NLR demonstrated significantly different result between two groups. These research results were consistent with several previously published studies. ^22,23^ On the other hand, the platelet count in this research was comparable between the groups of deceased and surviving patients. This was in contrast with the results of a meta-analysis that concluded by Lippi *et al.*, who showed that thrombocytopenia was associated with increasing severity risk and mortality of COVID-19. ^24^ Differences in pathophysiological mechanisms in each patient may lead to insignificant findings in this research. Many researchers have studied the changes in peripheral blood cell counts in COVID-19, and the results were that in infected patients, the white blood cell and neutrophil count increased, while the lymphocyte and platelet counts decreased. ^25^ In the other cases, coagulation abnormalities (prolonged PT and aPTT) and intravascular coagulopathy (DIC) were so correlated with low platelet count. ^26^ 

**Comment 8:**

 Thank you for your very helpful comment. We revised the manuscript in Discussion paragraph 6.

 D-Dimer in our study were measured using ELFA (enzyme linked fluorescent assay) method and cut off point 0.410 µg/ml. Several literatures showed different cut off point for D-Dimer. Poudel *et al.* , with 182 subjects, showed D-Dimer levels of 1.5 µg/ml were an optimal cut off point to predict mortality. While Zhang, 2020, with 343 subjects, shows D-Dimer level 2 µg/ml were an optimal cut off point. Guan, 2020 in China, with 1099 subjects, showed that non-survivors had a significantly higher D-dimer (median: 2.12 μg/ml) than that of survivors (median: 0.61 μg/ml). This result could be due to differences in measurement methods as disclosed by Favaloro *et al.* who stated several things regarding the measurement and reporting quality of D-dimers, such as the measurement method, cut-off value, or D-dimer unit [D-dimer unit (DDU)] can lead to different research results. ^30^

 Other factors in our study that can affect D-dimer level were our patients were older with median age 52, comorbidities such as cardiovascular disease and liver disease. Liver disease can affect D-Dimer elimination from blood. In this study D-dimer level was also measured on admission, very early in disease course. The half-life of D-dimer was approximately 8 hours therefore, serial measurement of D-dimer will shows more information

# Peer review: Reviewer response for version 1

**DOI:** 10.5256/f1000research.57221.r91679

**Published:** 2021-08-27

**Authors:** Andhika Rachman, Bayu Rumondor

**Affiliations:** 1 Department of Hematology and Medical Oncology, University of Indonesia, Jakarta, Indonesia; 2 Kebayoran Baru General Hospital Jakarta, South Jakarta, Indonesia

The aim of this study is to evaluate and analyze risk factors of death, hematological and coagulation factors for COVID patients at RSUD Dr. Soetomo Surabaya. The parameters used in this manuscript were Hb, WBC, Neu#, lim#, NLR, platelet count, aPTT, PT, D-dimer, and CRP. 

1. The statement "significantly different in the deceased group" might need to be rephrased to make it more clear.
2. In the discussions section under "survival analysis" it is stated that "leukopenia, leukocytosis, neutrophilia, high NLR, CRP and PT correlates with survival". This might confuse the readers. The writers might need to rephrase the statements to make it more in line with the abstract and with following statements.
3. The writers might need to detail the inclusion criteria used in the study. The writers stated that probable patients are included in the study, but the analysis is aimed more towards confirmed cases (the 217 subjects). In figure 1, it is indicated that 217 confirmed patients with complete data proceeded to statistical analysis.
4. The covid-19 degree of severity of the patients should be explained in more detail. This might help the reader to contextualize the term "non-ICU".
5. Kruskal Wallis method of analysis was not explained in the methods section.
6. The table 2 "comparison of the laboratory results in deceased and survived patients" may need some explanations. One example is that the writer used "above-normal result percentages".
7. Some grammatical errors might need to be addressed in the article.

Is the work clearly and accurately presented and does it cite the current literature?

Yes

If applicable, is the statistical analysis and its interpretation appropriate?

Yes

Are all the source data underlying the results available to ensure full reproducibility?

Partly

Is the study design appropriate and is the work technically sound?

Partly

Are the conclusions drawn adequately supported by the results?

Yes

Are sufficient details of methods and analysis provided to allow replication by others?

Partly

Reviewer Expertise:

immunology, cancer thrombosis and platelet

We confirm that we have read this submission and believe that we have an appropriate level of expertise to confirm that it is of an acceptable scientific standard, however we have significant reservations, as outlined above.

# Response

**Published:** 2021-08-28

**Authors:** Siprianus Ugroseno Yudho Bintoro

**Affiliations:** Airlangga University, Indonesia

Dear Reviewer,

 Thank you for reviewing and sending the feedback. We are going to revise it real soon.

 Warm regards,

 Research Team
